# Two classes of regulatory subunits coassemble in the same BK channel and independently regulate gating

**DOI:** 10.1038/ncomms9341

**Published:** 2015-09-21

**Authors:** Vivian Gonzalez-Perez, Xiao-Ming Xia, Christopher J. Lingle

**Affiliations:** 1 Department of Anesthesiology, Washington University School of Medicine, St. Louis, Missouri 63110, USA

## Abstract

High resolution proteomics increasingly reveals that most native ion channels are assembled in macromolecular complexes. However, whether different partners have additive or cooperative functional effects, or whether some combinations of proteins may preclude assembly of others are largely unexplored topics. The large conductance Ca^2+^-and-voltage activated potassium channel (BK) is well-suited to discern nuanced differences in regulation arising from combinations of subunits. Here we examine whether assembly of two different classes of regulatory proteins, β and γ, in BK channels is exclusive or independent. Our results show that both γ1 and up to four β2-subunits can coexist in the same functional BK complex, with the gating shift caused by β2-subunits largely additive with that produced by the γ1-subunit(s). The multiplicity of β:γ combinations that can participate in a BK complex therefore allow a range of BK channels with distinct functional properties tuned by the specific stoichiometry of the contributing subunits.

## Abstract (web summary)

Ion channels are often an assembly of proteins, but it is not clear if protein combinations have additive effects or function to prevent binding of other proteins. Here, the authors show that β and γ subunits can assemble into the same BK complex, and the constituents of the complex have an effect on its function.

## Graphical abstract

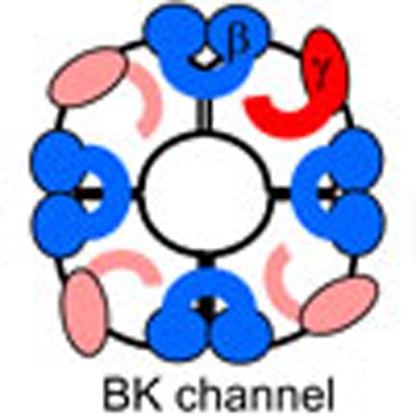

Proteomic^1^,^2^ and functional^2^,^3^,^4^,^5^,^6^ studies have revealed many partners that interact with BK channels, some of which are known to confer distinct tissue-specific functional properties on the BK pore-forming subunit. BK channels are dually activated by membrane depolarization and increases in intracellular [Ca^2+^] (refs 7, 8, 9). The minimal functional unit of a BK channel is a homotetramer of pore-forming α-subunits (Fig. 1a), each containing intrinsic voltage-sensing and Ca^2+^-sensing domains. However, BK channels may also contain any of two different families of regulatory proteins, β and γ, which help define tissue-specific functional properties of a BK complex. The four members of the β family (β1–β4) and the four members of the recently discovered γ family (γ1–γ4) differentially regulate BK function, influencing Ca^2+^-dependence of activation^2^,^5^,^6^,^10^,^11^, current inactivation^4^,^5^,^12^,^13^ and even pharmacology^5^,^14^.

β- and γ-subunits are unrelated proteins with very different predicted structural topology. β-subunits contain two transmembrane (TM) segments linked by an extracellular loop bridged by multiple disulfide linkages and intracellular C and N termini^15^; γ-subunits contain a single TM segment, a cytosolic C terminus, and an extracellular N terminus with a large leucine-rich repeat-containing motif^6^,^16^ (Fig. 1b). Specific structural information for β- and γ-subunits is not available. For β-subunits, potential positions of TM segments in relation to α-subunit TM segments have been proposed^17^,^18^,^19^ (Fig. 1c). Individual BK channels can contain 0–4 β-subunits, with each subunit contributing in an energetically independent fashion to shift BK gating^20^. For γ-subunits, both the position in the channel complex and the α:γ stoichiometry remain unknown. In heterologously expressed α+γ1 channels, the γ1-induced gating shift occurs in an all-or-none manner, consistent with an elementary functional unit of γ1 (for example, monomer, dimer and tetramer) being sufficient to produce the full effect^21^. Here to determine whether different types of regulatory subunits coassemble in the same BK channels, we take advantage of the distinctive functional properties conferred on BK channels by β2- and γ1-subunits. Using ensemble and single molecule approaches we report that γ1 and 1–4 β2-subunits can be simultaneously present in the same BK channel and independently contribute to modulation of BK function.

## Results

### β2- and γ1-subunits coassemble in the same BK channel

**Figure 1:**
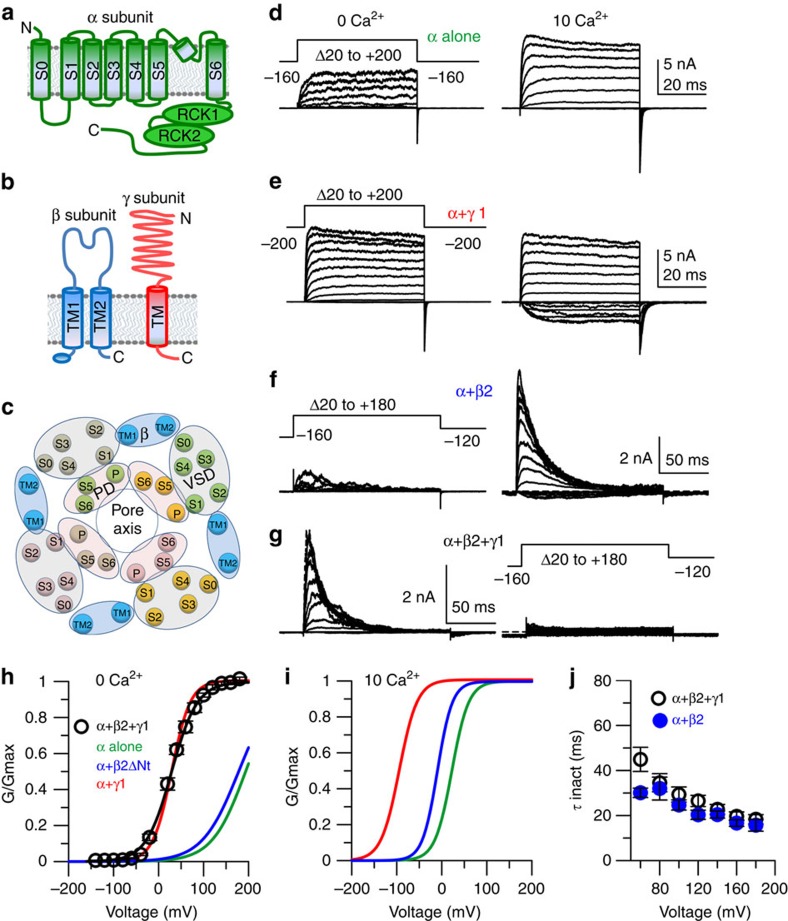
Coexpression of α+β2+γ1 subunits results in all BK channels containing both β- and γ-subunits. (**a**–**b**) Cartoons of the predicted topology of α-, β2- and γ1-subunits. The basic topology of β2- and γ1-subunits are shared with other members of their respective subunit families. (**c**) Schematic of deduced position of α- and β1-subunit TM segments in a BK channel complex viewed at the extracellular face^17^. Grey, pink and blue ellipses highlight voltage sensor domain (VSD), pore domain (PD) and β-subunit TMs, respectively. (**d**–**g**) Typical currents arising from (co)expression of BK subunits in oocytes: α alone, α+γ1, α+β2, or α+β2+γ1, respectively, at 0 (left) or 10 μM intracellular [Ca^2+^] (right); left and right currents were from the same patch using the same voltage protocol (depicted for each case). (**h**–**i**) Comparison among activation curves (*G*–*V*) at 0 Ca^2+^ or 10 μM of Ca^2+^, respectively, generated from sets of experiments as in panels (**d**–**g**). Notice the overlap between activation curves at 0 Ca^2+^ of α+γ1 and α+β2+γ1. *G*–*V* fits to single Boltzmann distributions yielded the following parameters: At 0 Ca^2+^, α-alone (*V*_h_=193±6 mV, *z*=0.7±0.02 *e*_*0*_, *n*=5); α+β2ΔNt (*V*_h_=182±4 mV, *z*=0.7±0.03 *e*_*0*_, *n*=5) ; α+γ1 (*V*_h_=29.0±4.7 mV, *z*=1.3±0.08 *e*_*0*_, *n*=7); α+β2+γ1 (*V*_h_=28.9±3.4 mV, *z*=0.9±0.04 *e*_*0*_, *n*=9). At 10 μM of Ca^2+^, α-alone (*V*_h_ =22.6±3 mV, *z*=1.4±0.12 *e*_*0*_, *n*=5); α+β2ΔNt (*V*_h_=−9.2±4.4 mV, *z*=1.6±0.87 *e*_*0*,_ *n*=5); α+γ1 (*V*_h_=−95.1±3.9 mV, *z*=1.4±0.05 *e*_*0*_, *n*=8). (**j**) Averages of *τ*_inact_ at voltages up to +180 for α+β2+γ1 at 0 Ca^2+^ (*n*=9) and α+β2 at 10 Ca^2+^ (*n*=6), respectively. All errors are s.e.m.

The γ1-subunit produces a remarkable negative shift of 120–140 mV in the voltage-range of BK channel activation either in the presence or absence of Ca^2+(2)^ (Fig. 1d,e,h,i). While BK channels composed of α alone require at least 10 μM Ca^2+^ to show appreciable open probability over physiologically relevant voltages, (Fig. 1h,i), α+γ1 channels show a similar probability in the total absence of intracellular Ca^2+^ (Fig. 1e,h). The ability of γ1 to shift BK gating in 0 Ca^2+^ contrasts with the absence of a gating shift produced by β2 (Fig. 1h) under the same conditions^22^.

**Figure 2:**
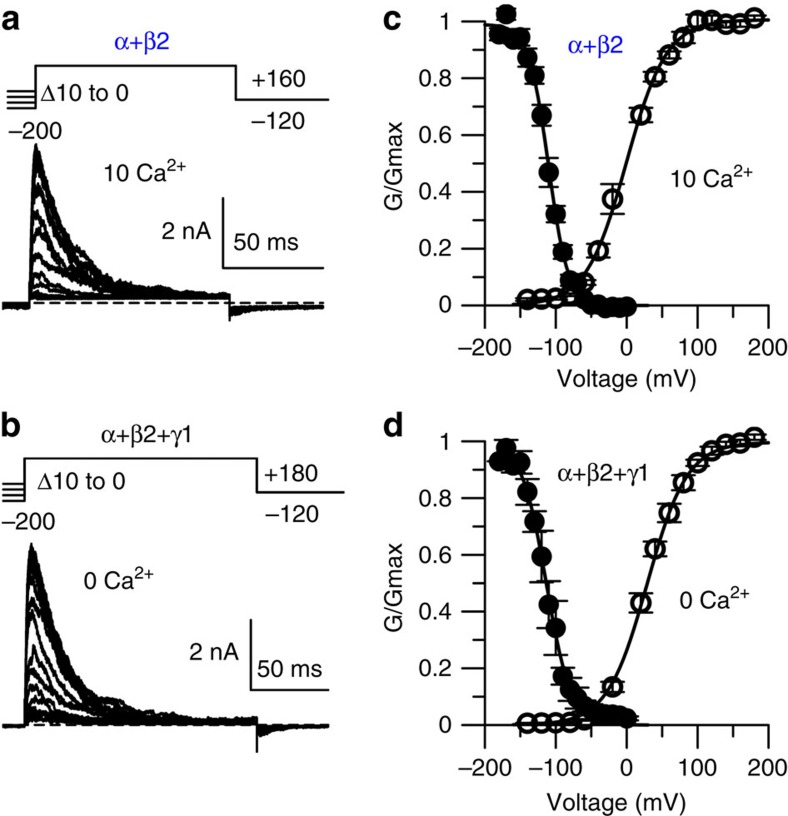
The voltage dependence of the fraction of channels available to activate also reflects the coassembly of β2- and γ1-subunits in the same channels. (**a**–**b**) Examples of the fractional availability of channels as function of the conditioning potential from oocytes injected with α+β2 (10 μM Ca^2+^) or α+β2+γ1 (0 Ca^2+^), respectively. Currents were evoked by the indicated protocols using conditioning potentials of 100 ms duration. (**c**–**d**) Curves of activation (open symbols) and fractional availability (filled symbols) as function of voltage obtained at 10 μM of Ca^2+^ for α+β2 or 0 Ca^2+^ for α+β2+γ1 channels, respectively, are plotted together. *G*–*V* fits to single Boltzmann distributions yielded the following parameters: α+β2 SS-inactivation (*V*_h_=−110.8±2.3 mV, *z*=1.81±0.11 *e*_0_, *n*=7) α+β2-activation (*V*_h_=−6.7±2.4 mV, *z*=0.96±0.31 *e*_*0*_, *n*=6); α+β2+γ1-SS-inactivation (*V*_h_=−113.5±5.3 mV, *z*=1.56±0.12 *e*_*0*_, *n*=4), α+β2+γ1-activation (also shown in Fig. 1h). All errors are s.e.m.

The most readily identifiable effect of the β2-subunit is essentially complete inactivation that occurs following channel activation (Fig. 1f). Inactivation arises from the cytosolic N terminus of the β2-subunit^4^,^5^,^23^. Since BK channels can contain 1–4 β2-subunits, each acting independently, single α+β2 channels exhibit one of four distinct inactivation rates^20^. The inactivation time constant (*τ*_inact_) therefore provides a measure of β2-subunit stoichioimetry within either a channel population or individual channels^20^. Although β2 produces little gating shift at 0 Ca^2+(23)^ (Fig. 1h), gating of α+β2 channels at 10 μM Ca^2+^ is shifted about 40 mV leftward compared with α channels^3^,^5^,^20^ which is more clearly observed using a non-inactivating β2 variant (β2ΔNt) (Fig. 1i). Thus, for α+β2 channels, voltage steps up to +180 mV only weakly activate BK currents at 0 Ca^2+^ (Fig. 1f, left), while 10 μM Ca^2+^ produces robust activation of an inactivating current (Fig. 1f, right), whose *τ*_inact_ approaches a limiting value of ∼20 ms (Fig. 1j), indicating an average of 4 β2-subunits/channel in the population^20^. Another physiologically relevant property of inactivating channels, the voltage dependence of steady-state inactivation (SS-inactivation), is also an indicator of the presence of β2-subunits. During exposure of α+β2 channels to 10 μM Ca^2+^, the availability of non-inactivated channels to open is very low at voltages above −50 mV (Fig. 2a,c) with half-channel availability (*V*_h_) at about −110 mV. Together, the distinctive effects of β2- and γ1-subunits provide useful signatures to verify the presence of β2 and γ1 in BK channels resulting from the coexpression of α+β2+γ1.

We therefore coexpressed γ1+β2 subunits with α at relative mole fractions of message that would produce full effects of either γ1 or β2 alone^20^,^21^. The coexpression of α+β2+γ1 subunits results in a prominent inactivating outward current even with 0 Ca^2+^ (Fig. 1g, left), similar to currents resulting from the expression of α+β2 subunits when activated by 10 μM Ca^2+^ (Fig. 1f). The complete inactivation of currents obtained after coexpression of α+β2+γ1 subunits indicates that essentially all channels contain β2-subunits. Furthermore, that in such patches the voltage dependence of activation at 0 Ca^2+^ is shifted more than −120 mV (Fig. 1h) is diagnostic for the presence of γ1. When the same patch is activated with 10 μM Ca^2+^, essentially no current is observed (Fig. 1g, right) which suggests that α+β2+γ1 channels are constitutively inactivated with 10 μM Ca^2+^ at −160 mV. Indeed, the fractional availability of α+β2+γ1 currents at 0 Ca^2+^ (Fig. 2b) exhibits a voltage dependence very similar to that observed with 10 μM Ca^2+^ for α+β2 currents (Fig. 2c–d). The markedly leftward-shifted steady-state inactivation curve of α+β2+γ1 channels also confirms that both β2- and γ1-subunits can coassemble in the same BK channels.

### β2 and γ1 occupy distinct positions in the BK channel complex

**Figure 3:**
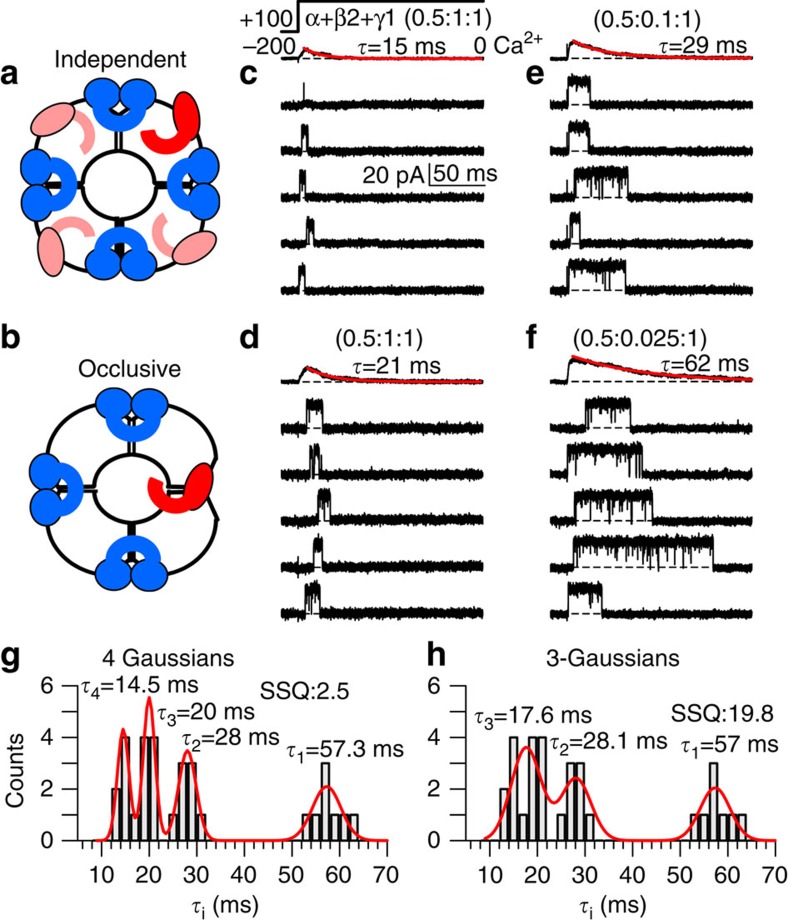
Single α+β2+γ1 channels simultaneously exhibit the maximal γ1-induced effect and up to four β2-subunits. (**a**–**b**) Cartoons representing two distinctive assembly models: where α, white; β2, blue; γ1, red. (**c**–**f**) Representative single channels obtained from various α:β2:γ1 injection ratios (given above each set of traces). For each example, five consecutive traces of current recorded at 0 Ca^2+^ are shown together with the ensemble current average from 100 total traces recorded in the same conditions. Red lines: fit of a single exponential with the indicated *τ*_inact_. (**g**–**h**) Grey bars represent the distribution of *τ*_inact_ from 31 single-channels (bin size: 2 ms). Red lines represent fits of the binned data to four- or three-component Gaussian distributions, respectively, both with the s.d. (*s*_*n*_) constrained such that *s*_1≥_ *s*_2≥_ *s*_3…_*s*_*n*_. Mean values (*τ*_*n*_) and sum of the squares of the errors (SSQ) obtained from each fit are indicated. The other parameters of each Gaussian component, amplitude (A_*n*_) and standard deviation, of the distributions are: four-component distribution (*A*_1_=2.1, *s*_1_=3.0; *A*_2_=3.5, *s*_2_=1.8; *A*_3_=5.6, *s*_3_=1.2; *A*_4_=4.3, *s*_4_=1.2) and three-component distribution (*A*_1_=2.05, *s*_1_=3.1; *A*_2_=2.4, *s*_2_=3.1; *A*_3_=3.6, *s*_3_=3.1).

We next wondered whether channels containing both β2+γ1 subunits can contain a full set of four β2-subunits. We imagined two kinds of assembly scenarios: (1) an independent model (Fig. 3a), where the presence of γ1-subunits does not hinder the ability of four β2-subunits to fully populate a BK channel (β2 and γ1 occupy different positions), or (2) an occlusive model (Fig. 3b), where the presence of γ1 excludes the assembly of β2-subunits (β2 and γ1 occupy overlapping positions). These two models can be tested by examination of the *τ*_inact_ arising from a set of single α+β2+γ1 channels obtained under conditions in which relative β2 subunit expression varies^20^. If the independent model is valid, γ1-containing inactivating single channels should exhibit four distinct *τ*_inact_ (ref. 20), while the finding of less than four inactivation behaviours supports the occlusive model. By keeping both α and γ1 constant but decreasing β2 in the RNA injection mix, we obtained a set of single channels that exhibit inactivation consistent with the presence of β2, but gating shifts consistent with the presence of γ1 (Supplementary Fig. 1). The *τ*_inact_ observed from 31 channels appeared to group into four behaviours (Fig. 3c–f). For channels that might potentially contain either three or four β2-subunits, the expected differences in mean inactivation time constant are not great. We therefore examined in detail the ability of three- and four-component Gaussian functions to fit the distribution. In evaluating fits of the distribution, we considered the impact of two criteria. First, since inactivation arises from independent inactivation domains^20^, the faster components should bear a simple arithmetic relationship to the slowest component. Second, the s.d. for any component, although perhaps not well-constrained by the data, should be smaller than for any slower components, such that *s*_1_≥*s*_2_≥*s*_3_≥*s*_4_. Various considerations in the fitting process are given in the Methods and in Supplementary Fig. 2. For the case that the s.d. are constrained as mentioned or where the mean values of each component are defined by the slowest component, the *τ*_inact_ distribution is better fit by a four-component than by a three-component Gaussian distribution (Fig. 3g,h, Supplementary Fig. 2), indicating that channels containing the γ1-induced effect can also contain one, two, three or four β2-subunits. In the case of the three-component Gaussian, if the constraint on s.d. is relaxed, the best fit is reached with the fastest component having the largest s.d. of any component, suggestive that this fast component in fact arises from two populations. The superior fit of the four-component Gaussian supports the existence of individual channels containing 4 β2 and at least one γ1-subunit indicating that both types of auxiliary subunits occupy independent positions in the channel complex (Fig. 3a).

### β2 and γ1 independently contribute to BK gating shifts

**Figure 4:**
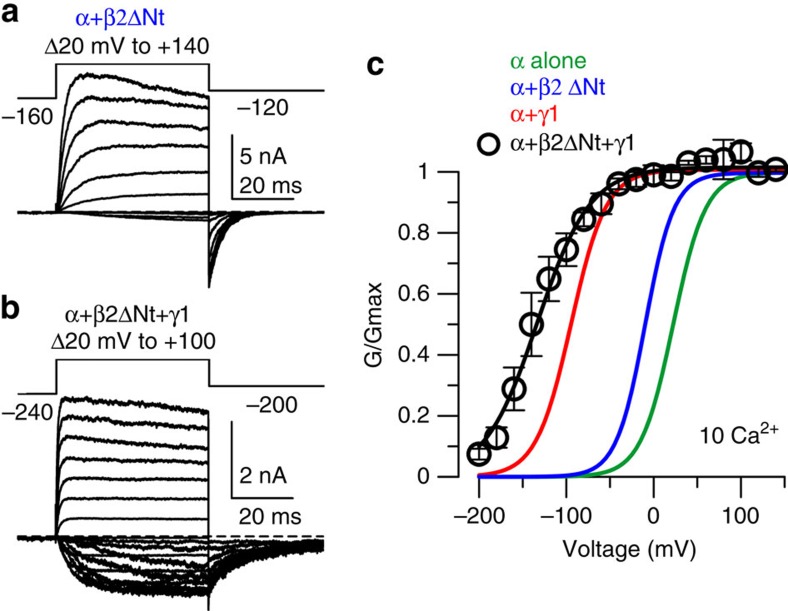
γ1 and β2 induced gating shifts are largely additive. (**a**–**b**) Currents arising from coexpression of α+β2-inactivation removed (β2ΔNt) (**a**) or α+β2ΔNt+γ1 (**b**) with 10 μM Ca^2+^. (**c**) Comparison of the gating shift obtained by β2 with or without the γ1 expression. β2 produces an approximately 30–35 mV shift with or without γ1. With 10 μM Ca^2+^, *V*_h_ for (α alone)=+22.6±3.4 mV, (α+γ1)=−95.1±3.9 mV, (α+β2ΔNt)=−9.2±4.4 mV and (α+β2ΔNt+γ1)=−134.7±8.8 mV (*n*=5); Δ*V*(β2)=31.8, Δ*V*(γ1)=117.7, Δ*V*(β2ΔNt+γ1)=157.3. All errors are s.e.m.

Both β2 and γ1 produce leftward shifts in BK gating, but their effects are likely mediated by different mechanisms. Whereas the γ1-effect can be explained by stronger coupling between the voltage-sensor movement and channel activation^2^, the effects of β2 appear more complex^22^,^24^,^25^,^26^. We asked whether γ1 and β2 effects might be additive or occlusive. For better elucidation of the β2 effects, we compared the gating shift resulting from the coexpression of γ1+β2ΔNt versus that produced by each construct separately when coexpressed with BK-α-subunits (Fig. 4). The *V*_h_ arising from the simultaneous presence of γ1 and β2ΔNt approximately reflects the sum of the independent effects of γ1 and β2ΔNt alone. These results indicate that, whatever the underlying molecular mechanism of the *V*_h_ shift produced by the γ1-subunit, it is predominantly energetically independent of that produced by the β2-subunit. Furthermore, there is no inhibitory allosteric coupling between the auxiliary subunits themselves.

### Other β-subunits also coassemble with γ1 in BK channels

Can other β-subunits also coassemble with γ1 in BK channels? An earlier report suggested that the presence of β1-subunits may occlude the ability of γ1 to produce its gating shift^2^. Since the overexpression of one subunit might influence the successful expression of another, we used proportions of RNA for each subunit similar to those used in testing γ1+β2 coassembly with α-subunits. Using a β1 construct in which its N terminus was replaced by the β2-N terminus (β1/β2Nt) so that inactivation reports the presence of β1, we found that all BK channels resulting from coexpression of α+β1/β2Nt +γ1 simultaneously contain both types of regulatory subunits (Supplementary Fig. 3).

## Discussion

The present work unambiguously shows that two different types of non-pore-forming regulatory subunits, β2 and γ1, can coassemble in the same functional BK channel and independently regulate channel function. The α+β2+γ1 combination generates a BK channel with novel functional properties, which subtly change depending on the stoichiometry of β2 in the multimeric complex. In a normal cellular environment, the simultaneous presence of β2+γ1 in BK channels might effectively remove them from availability for activation especially when intracellular calcium is increased. However, at 0 Ca^2+^, such channels would not be fully inactivated (Fig. 2d): the window of overlap between the fractional availability curve and the activation curve spans approximately −60 to −30 mV, a range corresponding to normal resting potentials in many types of cells, including neurons. This essentially defines a range of voltage over which any contributions of α+β2+γ1 channels might be dynamically regulated by inactivation. Given the large conductance of the BK channel, only a small fraction of the total BK population would have to undergo cycles of inactivation, recovery from inactivation and activation to have some influence on cell excitability. Although it is unknown whether β2+γ1 subunits are simultaneously present in any cell-type, reported message levels of both β2 and γ1-subunits in some tissues^11^,^27^ support the possibility that β- and γ-subunits will copartner in at least some cells. For any cells which may express both a β2- and a γ1-subunit, our results establish that these two distinct regulatory partners of BK channels can simultaneously and independently contribute to modulation of BK function and do not appear to hinder the assemble of each other into a channel. The possibility of coassembly would also likely apply to other members of the β and γ families, as supported by our results with β1+γ1 subunits. Our findings highlight not only the critical importance of defining the identity of protein partners in native multimeric complexes within a cell or a specific cell location, but also the importance of understanding how individual contributions of distinct regulatory components and their stoichiometry can define fundamental properties of the complex.

## Methods

### Constructs

Primary constructs were mouse α (SLO1) (ref. 9), human LRRC26 (γ1) (ref. 11) and human β2 (ref. 23). In some experiments β2ΔNt (the first 33 amino acids were removed from the β2-N-terminal) or β1/β2ΔNt (first 43 amino acids from the β2-N terminus replaced the first 11 amino acids of the β1-N terminus) were used.

### Oocyte expression

Stage IV *Xenopus laevis* oocytes were used for channel expression. The complementary RNAs (cRNAs) of all constructs were prepared at approximately 1 μg μl^−1^. For macroscopic recordings, cRNA mixes containing (molar ratio): mSLO1 alone, mSLO1+hβ2 (1:6.5), mSLO1+hLRRC26 (1:4) or mSLO1+hβ2+hLRRC26 (1:6.5:4), were diluted 1:5 before injection. For single channel recordings, mSLO1:hβ2:hLRRC26 (1: 0.65:4) and (1:0.16:4) were diluted 1:20–1:100 before injection. β2ΔNt or β1/β2ΔNt cRNAs were used at the same molar ratio as β2 in some experiments. Oocytes were used 2–5 days after injection, except for single channel recordings in which they were used 1–2 days after injection. Maintenance of frogs and isolation of oocytes following procedures approved by the Washington University in St Louis Institutional Animal Care and Use Committee.

### Electrophysiology

Borosilicate glass capillaries (1B150F-4, World Precision Instruments) were pulled to diameters resulting in resistances of 1–2 or 5–6 MΩ for macroscopic and single channel recordings, respectively. Pipettes were coated with Sylgard 184 (Dow Chemical Corp.) and fire-polished. Currents were recorded in the inside-out patch configuration using an Axopatch 200B amplifier (Molecular Devices) and the Clampex program from the pClamp software package (Molecular Devices). Gigaohm seals were formed in frog Ringer (in mM, 115 NaCl, 2.5 KCl, 1.8 CaCl_2_, 10 HEPES, pH 7.4) and, after patch excision, moved into flowing test solutions. The pipette/extracellular solution was (in mM): 140 K-methanesulfonate, 20 KOH, 10 HEPES, 2 MgCl_2_, pH 7.0. Test solutions of different Ca^2+^ contained^28^ 140 mM methanesulfonate, 20 mM KOH, 10 mM HEPES with pH adjusted to 7.0 (ref. 28). N-(2-hydroxyethyl)ethylenediamine-triacetic acid (HEDTA) was used for 10 μM Ca^2+^ and 5 mM ethylene glycol-bis(2-aminoethylether)-N,N,N′,N′-tetraacetic acid (EGTA) for 0 μM Ca^2+^solutions. The 10 μM Ca^2+^ solution was titrated to appropriate pCa with Ca-MES and calibrated against solutions of defined Ca^2+^ concentrations (World Precision Instruments) using a Ca^2+^-sensitive electrode. Test solutions were applied directly to patches via a large bore pipette tip containing multiple independent solution lines. All experiments were at room temperature (∼22–25 °C).

### Data analysis

Analysis were made using Clampfit (Molecular Devices). For non-inactivating currents, conductance values (G) were obtained from the tail currents, while for inactivating currents the peak current was used. *G*–*V* data sets were fitted with a single Boltzmann function: 
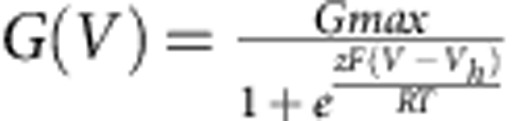
, where *V*_h_ represents the voltage of half activation and *z* is the valence of the voltage dependence. Notice that the α+γ1 injection ratio used (1:4 molar ratio) results in around 90% of BK channels expressed containing the full γ1-induced effect^21^, in which case the fit to a single Boltzmann distribution provides a good estimation of *V*_h_ and *z* for α+γ1 currents. Macroscopic inactivation time constants (*τ*_inact_) were obtained by fitting each current decay to a single exponential function.

All error estimates are SEM.

Single-channel traces were first processed using digital subtraction of leak and capacitive current defined from traces lacking any channel opening. *τ*_inact_ for each single channel was estimated from the ensemble current average of 50–100 identical sweeps recorded at 0 Ca^2+^. The histogram of *τ*_inact_ was generated using a bin size of 2 ms and the distribution was fitted to the sum of three or four components Gaussian function:




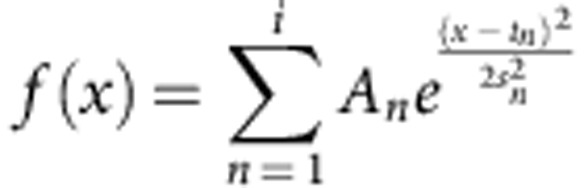




where *A*_*n*_, τ_*n*_ and *s*_*n*_ represent the amplitude, mean-*τ*_inact_ and s.d. of each component, respectively, with *n*=1 for the parameters of the slowest inactivating component, *n*=2 for the second slower and so on. Since each measured *τ*_inact_ represents the mean of an exponentially distributed population, the s.d. for the average of those grouped in the slowest component of the *τ*_inact_-distribution should be larger than for faster components, such that *s*_1≥_*s*_2≥_*s*_3≥_*s*_4_. Fitting to the sum of three or four Gaussian functions was first made allowing free variation of all fitting parameters. However, in both cases the rank of s.d. do not follow the expected criteria mentioned before: the four-component fit yields a very narrow third-component (very small *s*_3_), while the three-component fit results with the fastest component having the largest s.d. (*s*_3_) among all components (Supplementary Fig. 2a–b). Two factors may contribute to such deviations: first, that the numbers of entries in the histogram are insufficient to accurate define all aspects of each component and, second, that some components may actually arise from multiple components. We then constrained the s.d. in both cases to fulfil the expected criteria (*s*_1≥_s_2≥…_*s*_*n*_) (see results in Fig. 3g–h). Furthermore, we took advantage of the idea that inactivation results from the independent movement of each inactivation domain to the central cavity of the pore^20^. Based on this latter consideration, in some cases we also constrained all mean values of each component to be dependent on the slowest component (Supplementary Fig. 2c–d). All cases yield better fits using four than three-component Gaussian distributions.

### Chemicals

Salts and buffers were obtained from Sigma.

## Additional information

**How to cite this article:** Gonzalez-Perez, V. *et al*. Two classes of regulatory subunits coassemble in the same BK channel and independently regulate gating. *Nat. Commun.* 6:8341 doi: 10.1038/ncomms9341 (2015).

## Supplementary Material

Supplementary Information Supplementary Figures 1-3
