# Supplementary material for: Two classes of regulatory subunits coassemble in the same BK channel and independently regulate gating
Source: Nat Commun. 2015 Sep 21;6:8341. doi: 10.1038/ncomms9341 (PMC4578311; doi:10.1038/ncomms9341)
Supplement: Supplementary Information — Supplementary Figures 1-3 [file ncomms9341-s1.pdf]

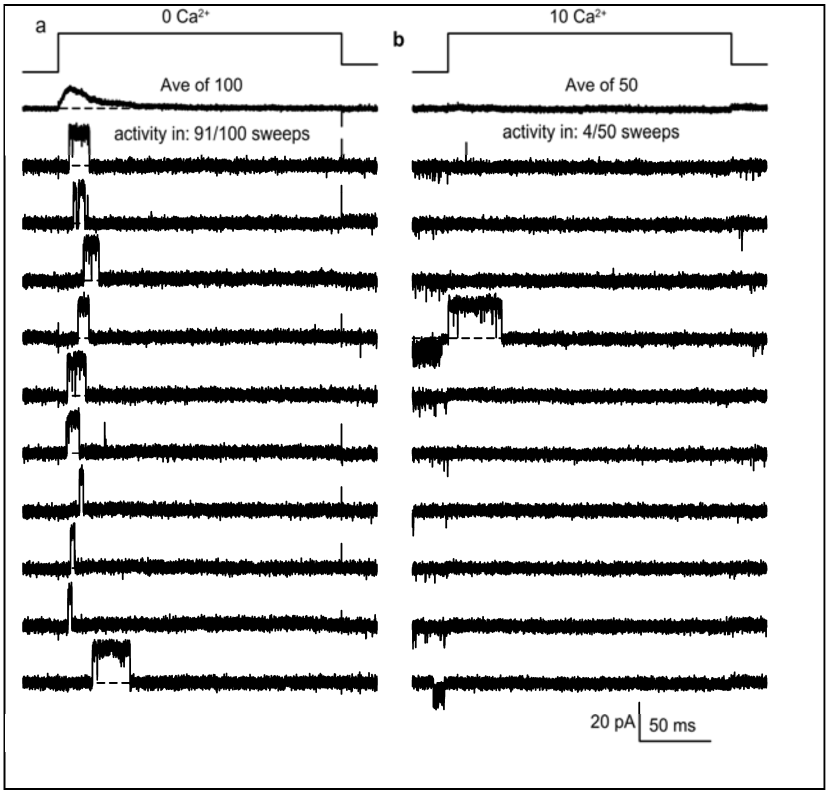

Supplementary Figure 1. Criteria that establish that inactivating single BK channels also contain γ1 subunits. For every single channel which exhibited inactivation, the presence of γ1 was confirmed by determination of the fraction of sweeps which exhibited a channel opening at either 0 or 10 Ca^{2+}μM, respectively, using a voltage step to +100 mV (depicted in the figure). Since γ1 shifts both the activation and the steady state inactivation curves by -120 mV, individual BK channels containing β2+γ1 behave like the example shown in **(a-b)**. **a,** At 0 Ca^{2+}, activation and then inactivating activity is observed in >90 % of sweeps (because Po (+100 mV) of γ1-containing channels is close to maximum). The first 10 of 100 sweeps recorded are shown together with the ensemble current average. **b,** For the same patch, at 10 μM Ca^{2+} channel activity is observed in <25% of sweeps (because at -200 mV channels are mostly inactivated prior to the depolarizing voltage step).The first 10 of 50 sweeps recorded at 10 μM Ca^{2+} are shown together with the ensemble current average.

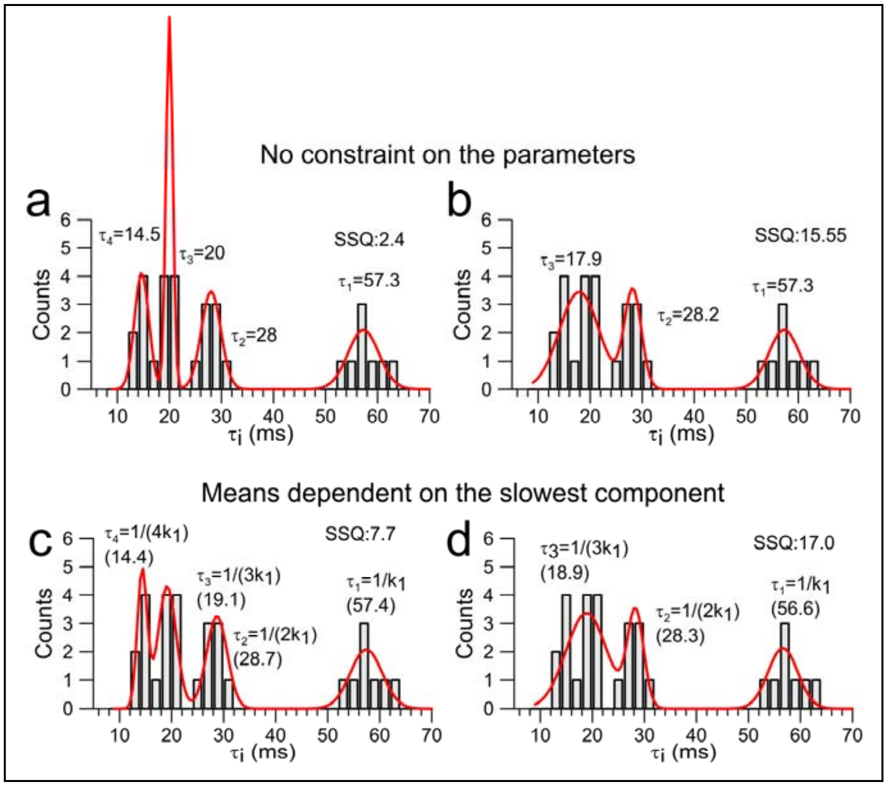

Supplementary Figure 2. Comparison between fitting of 4 or 3-component Gaussian distributions of measured τ_{inact}. a-b, Fits of 4 or 3-component Gaussian distributions, respectively, without constraint on any parameter. Mean values (τ_n) and sum of the squares of the errors (SSQ) obtained from each fit are indicated in the panels. The other parameters of each Gaussian component, amplitude (A_n) and standard deviation (s_n), of the distributions are: 4-component distribution (A_1= 2.1, s_1= 3.0; A_2= 3.5, s_2= 1.9; A_3= 12.5, s_3= 0.66; A_4= 4.1, s_4= 1.4) and 3-component distribution (A_1= 2.1, s_1= 2.9; A_2= 3.5, s_2= 1.6; A_3= 3.5, s_3= 3.8) where A_1 in each case represents the component with the longest measured τ_{inact}. Notice that in both cases the changes in standard deviation do not follow the idea that $s_1 > s_2 > s_3 > s_4$ mentioned in the Methods. Two factors may contribute to such deviations: first, that the numbers of entries in the histogram are insufficient to accurately define all aspects of each component and, second, that some components may actually arise from multiple components. **c-d,** Fits of 4 or 3-component

Gaussian distributions, respectively, where all means were constrained to depend on the slowest component. Notice that a 4-component distribution with dependent means (c) provides a better fit to the distribution than a 3-component distribution without any constraint (b), even when both fitting functions have the same number (9) of free parameters. The rest of parameters of the fittings shown in c-d are: 4-component distribution dependent on the slowest component (A_1= 2.1, s_1= 3.1; A_2= 3.3, s_2= 2.0; A_3= 4.3, s_3= 1.8; A_4= 4.9, s_4= 0.9) and 3-component distribution dependent on the slowest component (A_1= 1.0, s_1= 2.8; A_2= 0.84, s_2= 1.6; A_3= 2.1, s_3= 3.9). Notice that the expected rank of standard deviations ($s_1 > s_2 > s_3 > s_4$) is achieved with the 4-component fit simply from constraining the means to depend on the slowest component, something not achieved with the constrained-means 3-component fit.

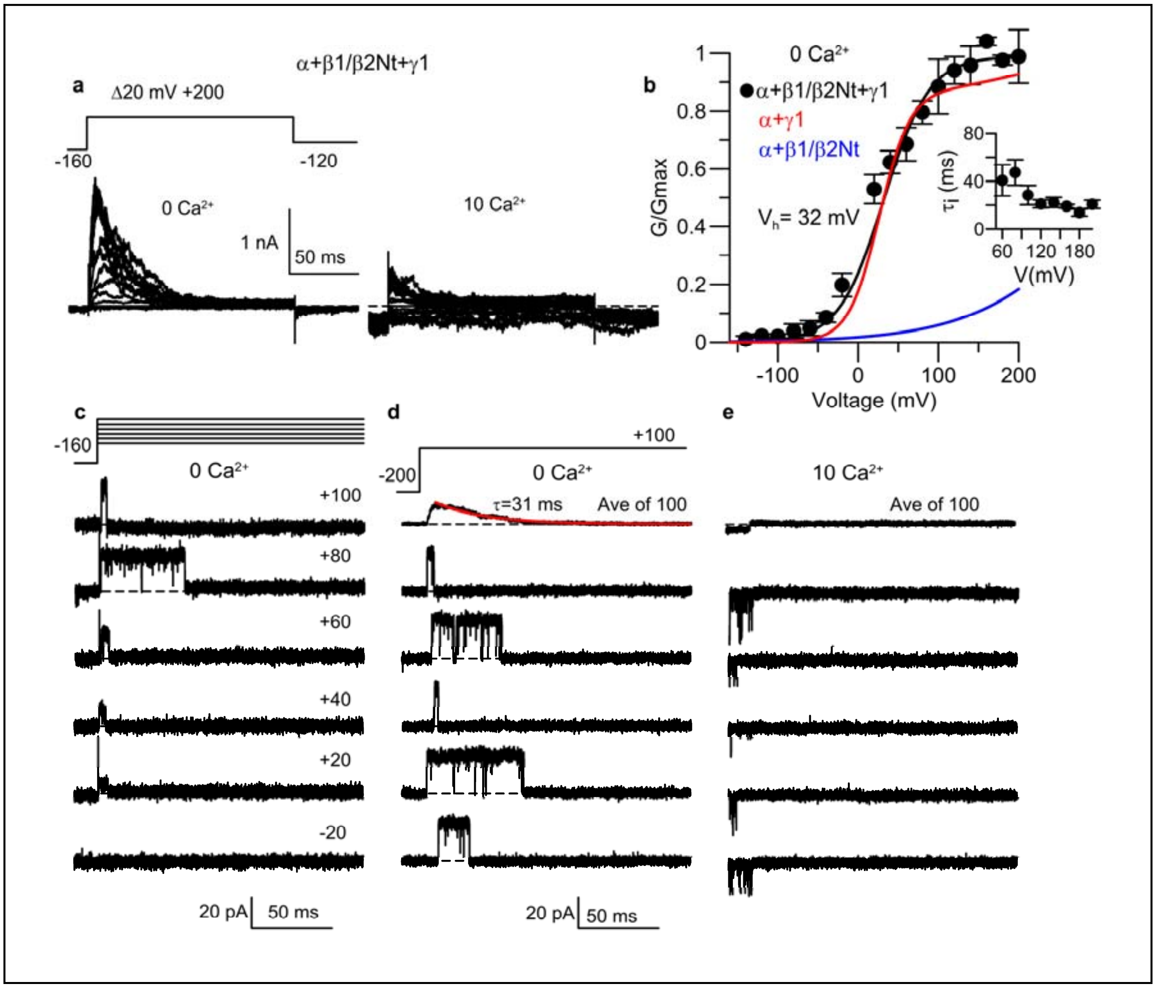

Supplementary Figure 3. β1 and γ1 also coassemble in the same channel. a, Representative currents arising from coexpression of α+β1/β2Nt+γ1, recorded in the same patch, at 0 (left) or 10 μM (right) intracellular [Ca^{2+}] using the protocol indicated on the top. **b,** Voltage-dependence of activation at 0 Ca^{2+} of α+β1/β2Nt+γ1 channels (V_h indicated, n=4) in comparison with those resulting from the separate coexpression of each regulatory subunit with α subunit. **inset,** Voltage-dependence of the τ_{inact} of α+β1/β2Nt+γ1 channels. **c-e**,Typical recordings from single channels resulting from coexpression of α+β1/β2Nt+γ1. **c,** A voltage protocol with increasing depolarizing steps every 20 mV applied at 0 Ca^{2+}. The corresponding voltages are depicted for each sweep shown. **d-e,** In the same patch as **(c)**, either 0 or 10 μM of Ca^{2+} were used to record

100 identical sweeps. The ensemble average and 5 consecutive individual sweeps are shown for each condition.
